# Perception of AI-generated smile versus real orthodontic treatment outcomes among dentists, students, and laypeople

**DOI:** 10.1038/s41598-026-41744-4

**Published:** 2026-03-21

**Authors:** Otso Tirkkonen, Gil Guilherme Gasparello, Sergio Luiz Mota-Júnior, Claudia Trindade Mattos, Marco Antonio Dias da Silva, Matheus Melo Pithon, Orlando Tanaka

**Affiliations:** 1 Research Unit of Population Health, University of Oulu, Oulu, Finland; 2 The Wellbeing Services County of North Ostrobothnia, Oulu, Finland; 3 Dentistry Department, Pontifícia Universidade Católica do Paraná – PUCPR School of Medicine and Life Sciences, Curitiba, PR Brazil; 4 Universidade Federal do Rio de Janeiro, Rio de Janeiro, Brasil; 5 Department of Orthodontics, Universidade Federal Fluminense, Niterói, Rio de Janeiro, Brazil; 6 Research Group of Teleducation and Teledentistry, Federal University of Campina Grande, Campina Grande, Brazil; 7 Brazilian Board of Orthodontics – BBO, Southwest Bahia State University - UESB, Jequié, Bahia Brazil; 8 Graduate Dentistry Program in Orthodontics, School of Medicine and Life Sciences, Pontifícia Universidade Católica do Paraná, R. Imaculada Conceição, 115, Curitiba, PR 80215-901 Brazil

**Keywords:** Artificial intelligence, Deep learning/machine learning, Orthodontic(s), Informatics, Esthetic dentistry, Health care, Mathematics and computing

## Abstract

In orthodontics, the increasing use of AI-generated smile images in patient communication raises ethical and practical concerns about user perception and misinterpretation of these visuals. This cross-sectional, non-probabilistic sample study evaluated the ability of dentists, dental students, and laypeople to distinguish between real and AI-generated orthodontic smile images, and their perceived attractiveness. The final sample consisted of 288 participants, (63.4% female; mean age = 32.4 years) including 76 dentists, 63 dental students, and 149 laypeople. Each participant was presented with three clinical scenarios, mild dental misalignment, midline diastema, and moderate anterior crowding, and viewed randomized sets of images depicting pre-treatment, real post-treatment, and AI-generated smiles. For each image, participants indicated whether they believed it was AI-generated or real and rated its aesthetic appeal using a visual analog scale ranging from 0 to 100. Data were analyzed using descriptive statistics and diagnostic performance metrics (accuracy, sensitivity, specificity, PPV, and NPV), with attractiveness ratings compared between AI-generated and real images. Sensitivity for identifying AI-generated images was low across all groups (< 50%), while specificity for recognizing real images was high (> 87%). Dental students achieved the highest overall accuracy (72.7%), followed by laypeople (66.3%) and dentists (62.6%). AI-generated smiles were consistently rated as significantly more attractive than real outcomes by all groups (mean VAS: 78.8 vs. 37.9; *p* < 0.001). AI-generated smile images were less accurately identified and more aesthetically pleasing than real clinical outcomes compared to real post-treatment outcomes, which were more consistently recognized across all participant groups, regardless of treatment type.

**Supplementary Information:**

The online version contains supplementary material available at 10.1038/s41598-026-41744-4.

## Introduction

 Recent advancements in Generative Artificial Intelligence (Gen-AI) have rapidly transformed digital content creation, allowing users to generate high-quality outputs, such as images, text, and music, from simple textual prompts^1^. Among these, text-to-image models have gained particular prominence, providing accessible tools capable of producing photorealistic visuals based on user-defined descriptions^2–4^. Advanced platforms such as DALL·E 2, Midjourney, and Stable Diffusion have contributed to the widespread diffusion of this technology by offering user-friendly interfaces^5,6^. As a result, AI-generated images are increasingly visible in domains ranging from digital art and marketing to journalism and personal content creation^6–8^.

The integration of multimodal capabilities in tools like ChatGPT has broadened public access to generative image technologies^9^. By combining natural language processing and image generation in a single interface, ChatGPT allows users to create visuals without technical expertise^10^. Unlike other platforms that require third-party tools or prompt engineering, it offers a seamless, dialogue-based experience^5,6^. This accessibility is particularly valuable in healthcare communication, where realistic images can be generated by non-experts. In 2025, the DALL·E 3 model within ChatGPT brought enhanced photorealism, anatomical precision, and better prompt interpretation, expanding its utility in clinical and professional contexts.

Despite their growing presence in public and professional communication channels, the reception of AI-generated images by different user groups remains insufficiently explored. Previous research in the Human-Computer Interaction field has predominantly focused on the artistic and creative aspects of AI-generated imagery, investigating user practices and aesthetic judgments in comparison with human-made artwork^11–13^. However, the extent to which laypeople and professionals can distinguish between AI-generated and human-generated images, particularly outside artistic contexts, remains an open research question.

This gap is especially relevant in fields such as dentistry, where visual representation of treatment outcomes plays a critical role in shaping patient expectations and clinical communication. In orthodontics, before-and-after smile photographs are commonly used for educational, promotional, and diagnostic purposes^14^. The viral dissemination of such content on social networks amplifies the influence of idealized dental aesthetics, potentially blurring the line between realistic clinical outcomes and digitally fabricated results.

Moreover, psychological theories such as the “uncanny valley”^15^ suggest that hyper-realistic yet imperfectly human-like representations may evoke discomfort or unease. At the same time, people may anthropomorphize AI systems, attributing human-like capabilities or biases to them^16,17^. Understanding whether and how different audiences interpret AI-generated dental images is therefore essential to inform responsible clinical communication and ethical deployment of such technologies in healthcare settings. By comparing AI-generated and real orthodontic treatment outcomes, initial evidence can be gathered on the potential applications of AI-generated visual outcomes in dentistry in the future. Moreover, the present study aimed to examine how dentists, dental students, and laypeople perceive AI-generated smile images in comparison with real orthodontic treatment outcomes. Specifically, this research sought to determine (i) whether participants could correctly identify whether an image was AI-generated or real, and (ii) which images were perceived as more attractive.

## Methods

### Study design

This cross-sectional, non-probabilistic observational study evaluated participants’ ability to distinguish between AI-generated and real orthodontic treatment outcome smile images, as well as their perceived attractiveness. This study was approved by the Ethics Committee of the Pontifícia Universidade Católica do Paraná (PUCPR), Brazil (2235302). All participants provided informed consent prior to participation, and the study was conducted in accordance with the Declaration of Helsinki and relevant institutional guidelines. Participation was voluntary, anonymous, and devoid of identifiable data. Informed consent was obtained from all participants and from individuals whose anonymized smile images were used, authorizing their use for research and educational purposes. The study complied fully with the Declaration of Helsinki and its amendments.

### Image preparation

Three anonymized pre-treatment smile photographs were selected from the patient archive of the private clinic. The selection process was conducted by three orthodontic specialists. The experts selected by consensus, three representative cases reflecting common clinical presentations: (1) mild dental misalignment, (2) a midline diastema, and (3) moderate anterior crowding. All photographs had been originally captured using a Canon EOS Rebel T7 DSLR camera equipped with a 100 mm macro lens. The images were taken in front of a neutral white background, in natural head position, with the camera placed at a distance of 1.5 m, at the height of the patient’s mouth. Patients were instructed to maintain a natural smile with lips slightly parted, allowing full visibility of the anterior teeth without forced stretching. All images were framed in portrait orientation, including the lower third of the face. The image were:

1. A patient with mild dental misalignment.
2. A patient with a midline diastema.
3. A patient with moderate anterior crowding.

For each case, three images were prepared:

- The original pre-treatment smile photograph.
- The post-treatment smile photograph following completion of orthodontic treatment with fixed appliances.

An AI-generated smile image was created using the paid version of ChatGPT (Version GPT-4o, OpenAI, San Francisco, United States) on April 19, 2025. A newly registered account was used exclusively for image generation. Each of the pre-treatment images was individually uploaded into the system to guide the generation process, using the only single prompt: ‘Make a realistic perfect smile’ (Supplementary material 1).

The number of stimuli was intentionally limited to minimize cognitive overload and participant fatigue, a strategy widely recommended in visual perception research to enhance attentional engagement and response consistency. Cases were chosen to represent comparable clinical presentations, avoiding extreme anatomical variations that could bias aesthetic evaluation.

**Fig. 1:**
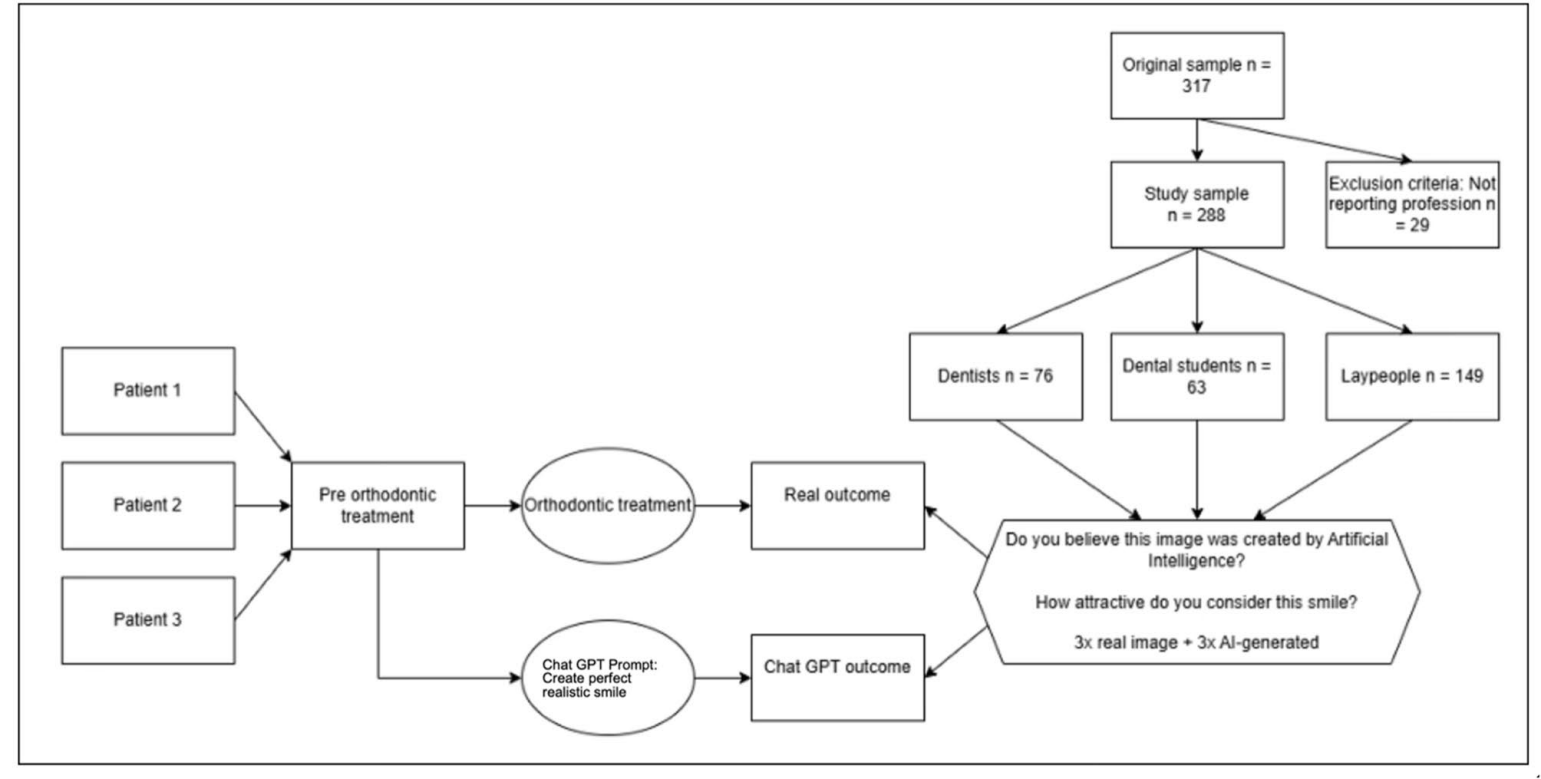
Study flowchart illustrating the methodology for generating and smile images.

The process is explained in Fig. 1.

### Sample calculation

Sample size calculation was conducted considering the Brazilian population as the reference population. Assuming an infinite population, a 95% confidence level, and a margin of error of 6%, the required minimum sample size was calculated using the standard formula for proportions. Based on these parameters, the minimum required sample size was determined to be 267 participants. The original sample included 316 responses. After applying the exclusion criteria (participants under 18 years of age and those who did not report their profession) the final sample consisted of 288 participants. This number exceeded the minimum required sample size.

#### Participants

The final sample consisted of 288 participants mean age of 32.4 years (SD = 12.0). The sample was predominantly female, comprising 182 women (63.4%) and 105 men (36.6%). One not reported, divided into three distinct groups.

- Practicing dentists (*n* = 76),
- Undergraduate dental students (*n* = 63),
- Laypeople without formal dental training (*n* = 149).

Participants were recruited via professional networks, dental schools, and general social media announcements to ensure diversity in professional background and familiarity with dental aesthetics.

### Data collection

Data collection was conducted using the Qualtrics (QualtricsTM, Provo, Utah, United States) online survey platform between April 26 and May 8, 2025, targeting participants residing in Brazil. Participants were presented with three sets of images, each set consisting of a pre-treatment smile photograph, the corresponding post-treatment photograph, and an AI-generated image. The presentation order of the images within each set was randomized to minimize potential order effects and response bias. All responses were submitted electronically through the Qualtrics platform within the defined data collection period. Pre-treatment images were excluded from the statistical analysis, as the primary aim of the study was to compare actual post-treatment outcomes with AI-generated images.

For each image, participants answered the following two questions:

1. Identification task:
2. *“Do you believe this image was created by Artificial Intelligence?”*
3. Participants responded using a binary choice (Yes/No).
4. Attractiveness Rating:
5. *“On a scale from 0 to 100, how attractive do you consider this smile?”*
6. (0 = Not attractive at all; 100 = Extremely attractive).

In addition to the image evaluation task, participants were asked to provide demographic information to characterize the study sample. The collected data included age, sex, educational level, employment status, marital status, and geographical region of residence within Brazil. Participants were also asked to report their professional background, specifying whether they were practicing dentists, dental students, or laypeople without formal dental education.

### Data analysis

All responses collected through the Qualtrics platform were exported in Microsoft Excel® for Microsoft 365 (Microsoft Corporation, Redmond, WA, USA) and subsequently imported into R software, version 4.4.0 (R Core Team 2025. R: A Language and Environment for Statistical Computing. R Foundation for Statistical Computing, Vienna, Austria) for analysis. Descriptive statistics, including absolute and relative frequencies, means, and standard deviations, were calculated to characterize the sample and summarize the response patterns. To assess differences in participants’ performance of identifying AI-generated and real images we employed several metrics including accuracy, sensitivity, specificity, positive predictive value (PPV) and negative predictive value (NPV). Accuracy was reported with a 95% confidence interval (CI). Sensitivity was utilized to evaluate the performance of correctly identifying AI-generated images, while specificity was utilized to assess the performance of identification of real images. For the calculation of PPVs and NPVs, AI-generated images were classified as positive cases and real images as negative cases. Additionally, attractiveness ratings between AI-generated and real images were compared. Generalized Estimating Equations (GEE) were used to analyze participants’ accuracy in identifying AI-generated images, accounting for repeated measures within individuals. Age and sex were incorporated as covariates to adjust for potential confounding variables.

The following statistical tests were applied: Pearson’s Chi-Square Test to evaluate associations between categorical variables, particularly participants’ ability to correctly identify AI-generated and real images between groups. Kruskal–Wallis Test to compare attractiveness ratings (0–100 scale) across participant groups, given the ordinal distribution of the data and potential deviations from normality. The Mann–Whitney U test was used to compare the AI-generated and real orthodontic treatment outcome attractiveness among outcomes. To analyze the distribution of attractiveness ratings, we employed kernel density estimation (KDE) using the geom_density function in the ggplot2 package^18^. All statistical analyses adopted a significance threshold of *p* < 0.05.

### Pilot study

A pilot study was conducted with 40 participants (mean age = 32.6 years, SD = 8.4) to assess the test-retest reliability of the questionnaire. The sample consisted of 12 dentists, 14 dental students, and 14 laypeople. Each participant completed the same set of questions twice, with a 20-day interval between sessions. The test-retest reliability was found to be excellent for the VAS variable (Intraclass Correlation Coefficient = 0.88), and substantial agreement was observed for the binary AI identification item (Cohen’s Kappa = 0.89, *p* < 0.001). No significant differences were found between the two time points for either variable (paired t-test for VAS: t = 0.65, *p* = 0.52; McNemar’s test for binary variable: *p* = 0.41). The pilot data were not included in the final study sample because participants could be identified through unique matching codes, whereas all responses in the final sample were required to be fully anonymous.

## Results

From the 288 responses, the majority of respondents (51.7%, *n* = 149) reported having no professional background in dentistry or orthodontics, representing the layperson group. Nevertheless, the sample included a substantial proportion of practicing dentists (26.4%, *n* = 76) and dental students (21.9%, *n* = 63), allowing for comparisons between professional and non-professional perceptions. Participants represented a wide range of ages (mean = 32.4 years, SD = 12.0) and educational levels, with most having completed at least secondary education. Regarding gender distribution, the sample was predominantly female (63.4%, *n* = 182). Participants were recruited from all major regions of Brazil, with the largest proportion residing in the Southern region (62.9%, *n* = 180).

Regarding the distribution of participants’ responses when asked to classify smile images as either AI-generated or real, dentists correctly identified 39.1% of AI-generated images, while dental students correctly identified 48.9% of AI-generated images. Lay participants correctly identified 41.2% of AI-generated images. No statistically significant difference was observed between the groups regarding their ability to correctly identify AI-generated images (χ^2^, *p* = 0.111). However, a statistically significant difference was found among the groups in correctly identifying real orthodontic treatment outcomes (χ^2^, *p* < 0.001). Although all groups showed high accuracy in identifying real teeth images, dentists correctly identified them in 87.8% of cases, dental students in 98.2%, and laypeople in 93.0%. The results are presented in Table 2.

**Table 1:** Characteristics of study population.

Variable name	% (*n*)
Education	
High school diploma or equivalent	34.7% (100)
Undergraduate degree	25.7% (74)
Postgraduate degree (specialization)	20.8% (60)
Postgraduate degree (Master’s or doctorate)	18.8% (54)
Not reported	0
Sex	
Male	36.6% (105)
Female	63.4% (182)
Not reported	1
Employment	
Retired	1.39% (4)
Self-employed or entrepreneur	28.5% (82)
Unemployed or temporarily laid off	2.08% (6)
Employed in a permanent/full-time position	29.9% (86)
Full-time student	28.5% (82)
Full-time student and part-time employee	6.60% (19)
Other	3.13% (9)
Not reported	0
Profession	
Dentist	26.4% (76)
Dental student	21.9% (63)
Laypeople	51.7% (149)
Not reported	0
Marital status	
Married	38.5% (111)
Divorced or separated	4.17% (12)
Single	56.9% (164)
Widowed	0.347% (1)
Not reported	0
Region	
Central-west	9.44% (27)
Northeast	10.8% (31)
North	1.40% (4)
Southeast	15.4% (44)
South	62.9% (180)
Not reported	2
Age (mean(sd))	32.4 (12.0)
Not reported	3

**Table 2:** Confusion matrixes of distinguishing real and AI-created images stratified by clinical experience.

		Dentist	Dentist	Dental Students	Dental Students	Laypeople	laypeople	Chi square
		AI	Real	AI	Real	AI	Real	
Guess	AI	**39.1% (86)**	**(12.2%) 25**	**48.9% (89)**	**1.76% (3)**	**41.2% (180)**	**7.04% (29)**	***P*** ** = 0.111**
Guess	Real	**60.9% (134)**	**87.8%( 180)**	**51.1% (93)**	**98.2% (167)**	**58.8% (257)**	**93.0% (383)**	***P*** ** < 0.001**
	No response	8	23	7	19	10	35	
Total		228	228	189	189	447	447	

Statistical significance at p .05.

Dental students succeeded best distinguishing real and AI-created teeth with accuracy of 0.727 (95% CI 0.678–0.773). Their sensitivity was 0.489 reflecting poor ability to identify AI-created teeth. However, their specificity of 0.982 demonstrated an excellent ability of identifying real teeth. Dentists had the worst overall performance distinguishing real and AI-created teeth in terms of accuracy, sensitivity and specificity. The same trend of poor sensitivity and high specificity was observed among dentists and others. Additionally, when opting for an answer of AI created teeth dentists were incorrect most with the Positive Predictive Value (PPV) of 0.775 (Supplementary material 2).

Participants correctly identified 45.8% of AI-generated images in the mild dental misalignment condition (0.458 sensitivity), 40.6% in the midline diastema condition, and 40.4% in the moderate crowding condition with sensitivity of 0.404. No significant differences were found across conditions in identifying AI-generated (*p* = 0.348) or real images (*p* = 0.841) (Table 3). Although not statistically significant, mild misalignment showed slightly better sensitivity. However, sensitivity remained low across all conditions, indicating difficulty in detecting AI-generated images. In contrast, high specificity (0.921–0.935) was observed in all conditions, reflecting consistent recognition of real outcomes. PPV and NPV were similarly stable, with mild misalignment yielding the highest values (PPV 0.884, NPV 0.612) (Supplementary material 3). GEE analyses adjusting for age and sex demonstrated that these variables were not significantly associated with participants’ accuracy in identifying AI-generated images (all *p* > 0.05), indicating that the differences observed between groups were independent of demographic characteristics.

**Table 3:** Confusion matrixes of distinguishing real and AI-created images stratified by treatment type.

		Mild dental misalignment	Mild dental misalignment	Midline diastema	Midline diastema	Anterior crowding	Anterior crowding	Chi square
		AI	Real	AI	Real	AI	Real	
Guess	AI	**45.8% (130)**	**6.54% (17)**	**40.6% (113)**	**7.87% (21)**	**40.4% (112)**	**7.31% (19)**	***P*** ** = 0.348**
Guess	Real	**54.2% (154)**	**93.5% (243)**	**59.4% (165)**	**92.1% (246)**	**59.6% (165)**	**92.7% (241)**	***P*** ** = 0.841**
	No response	4	28	10	21	11	28	
Total		288	288	288	288	288	288	

Statistical significance at p .05.

**Fig. 2:**
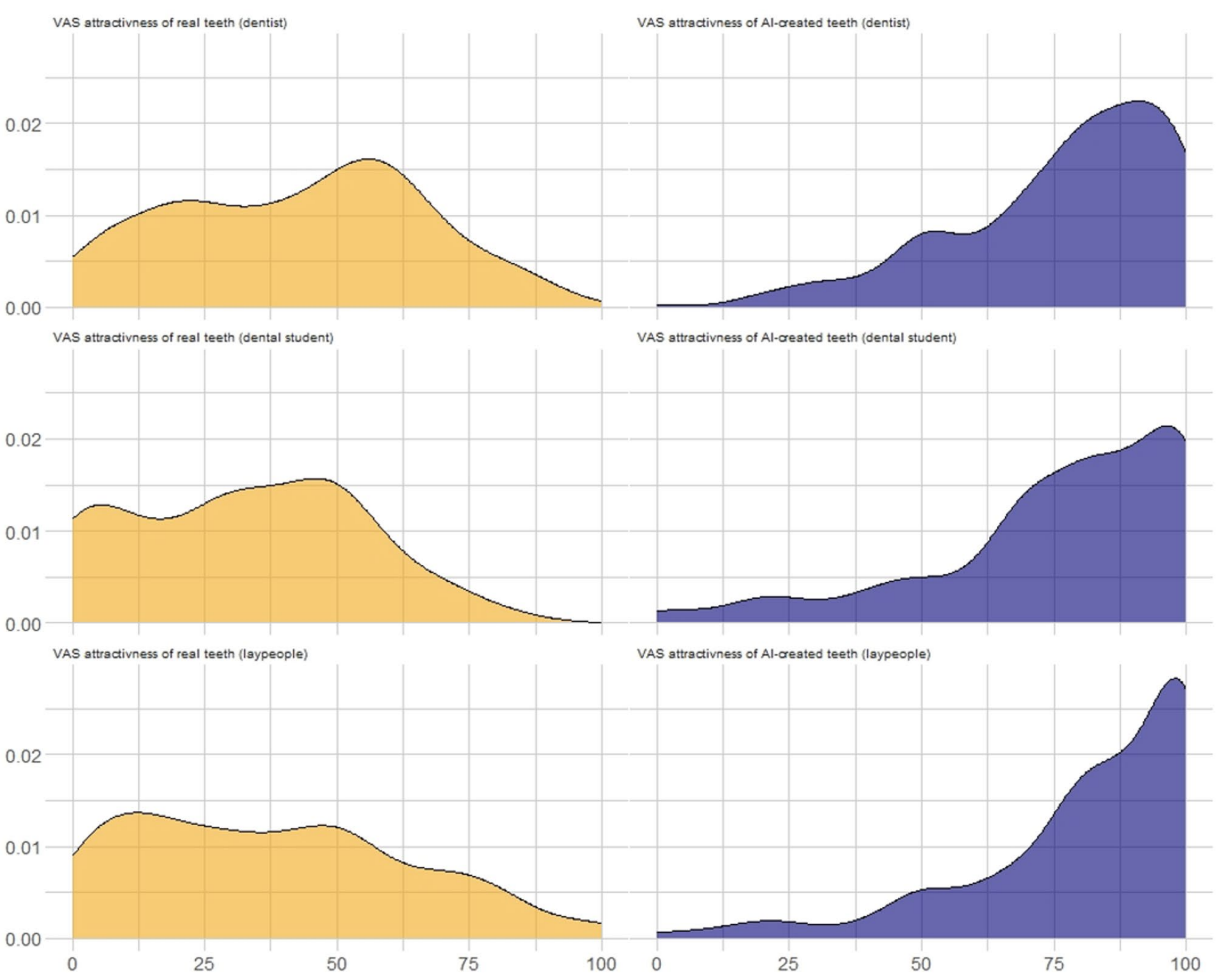
Kernell plots of dentists, dental students and layover people evaluating the attractiveness of AI-created and real orthodontic treatment outcomes. AI-created outcomes are marked with navy-blue and real outcomes as yellow. Visual attractiveness was evaluated with VAS scale (rating 0–100).

Overall, AI-generated smiles were rated significantly more attractive than real post-treatment outcomes, with mean VAS scores of 78.8 (SD = 22.0) for AI-generated images compared to 37.9 (SD = 24.9) for real orthodontic results (*p* < 0.001). Dentists rated the real post-treatment smiles with a mean VAS score of 42.6, while the corresponding AI-generated smiles received a significantly higher mean score of 76.6. Similarly, dental students assigned mean scores of 32.4 for real outcomes and 75.9 for AI-generated images, whereas laypeople rated the real smiles at 37.8 and the AI-generated ones at 81.1, the difference was statistically significant for all groups (*p* < 0.001). This preference for AI-generated smiles was consistent across all participant groups, including dentists, dental students, and laypeople (Fig. 2). The distribution plots show that high attractiveness ratings (VAS > 75) were frequent for AI-generated images across all groups, whereas such high ratings were rarely given to real treatment outcomes. Conversely, low attractiveness ratings (VAS < 25) were common for real orthodontic outcomes but nearly absent for AI-generated smiles, indicating a systematic tendency to favor the digitally created “perfect” smiles over real clinical results (Fig. 2).

## Discussion

This study revealed notable challenges in participants’ ability to accurately distinguish between AI-generated and real clinical images. All participant groups showed low sensitivity, below 50%, in identifying AI-generated content, confirming the sophistication of current generative AI models in producing highly realistic outputs that blur the boundary between authentic and artificial imagery, remarkably, this level of perceptual ambiguity was observed despite the use of a simple, non-optimized prompt and an easily accessible generative AI tool, highlighting the advanced state of AI capabilities even under minimally guided conditions. Interestingly, dental students demonstrated the highest specificity in identifying real clinical outcomes, possibly reflecting a younger generation more connected to AI features and recent academic exposure to diagnostic training^14^. However, their equally low sensitivity in identifying AI-generated images emphasizes a broader vulnerability across all experience levels, raising concerns about potential miscommunication in patient consultations and misleading interpretations in health-related digital media.

Historically, the Turing Test^19^ has served as a conceptual benchmark for determining whether a machine can imitate human behavior to the point of being indistinguishable from a human. Building on this principle, recent research has developed various methods to assess whether people can reliably recognize AI-generated content in different domains, including language, images, and multimedia^20–22^. Extending this evaluation to healthcare communication, the concept of perceptual thresholds, where AI-generated visuals are either accepted as authentic or recognized as artificial, emerges as a critical framework. Understanding these thresholds is essential for guiding ethical standards and best practices in the use of AI-generated imagery within patient communication, professional education, and digital health marketing.

Further analysis across different clinical scenarios, including mild dental misalignment, midline diastema, and moderate anterior crowding, revealed no significant variation in participants’ classification performance. While participants showed marginally higher sensitivity in detecting AI-generated images of mild misalignment, this difference was not statistically significant, reinforcing the finding that AI-generated visuals are challenging to detect across varying clinical presentations^11,23,24^. The consistently high specificity across all conditions suggests that participants were more confident in identifying real outcomes, likely influenced by a perceptual bias toward natural imperfections as indicators of authenticity^25,26^. This perceptual bias, however, did not prevent participants from overwhelmingly favoring AI-generated smiles in aesthetic evaluations, which were rated as significantly more attractive than real orthodontic outcomes across all groups. Such results align with broader research indicating that exposure to digitally enhanced content may shape unrealistic expectations of aesthetic outcomes^12,27^.

The present findings are consistent with growing evidence that humans struggle to reliably distinguish AI-generated images from authentic photographs. Prior research has demonstrated substantial misclassification rates and suggests that demographic characteristics such as age and gender do not significantly influence detection ability^28^. Moreover, human performance has been shown to be at or near chance levels, with synthetic faces sometimes perceived as more real and trustworthy than genuine ones. These perceptual limitations reinforce concerns that increasingly realistic generative models may challenge visual authenticity judgments across diverse populations. Importantly, the adjusted GEE models demonstrated that age and sex were not significant predictors of participants’ ability to correctly classify AI-generated images. This finding suggests that the difficulty in distinguishing synthetic from real orthodontic outcomes may reflect a generalized perceptual limitation rather than an effect restricted to specific demographic groups. The relatively homogeneous age distribution of the sample may have reduced variability and limited the detection of subtle age-related differences; however, the persistence of group effects after adjustment strengthens the interpretation that professional background, rather than demographic characteristics, plays a more relevant role in image evaluation. In addition, these findings carry implications that extend beyond professional practice into the wider digital ecosystem. The growing presence of AI-generated content on social media and digital platforms contributes to a media environment where authentic and artificial visuals are increasingly indistinguishable^12,29^. This not only complicates informed decision-making but also risks reinforcing unattainable beauty standards and propagating misinformation. Exposure to idealized, AI-generated imagery may negatively impact users’ self-perception, body image, and trust in healthcare services^30–33^. Addressing these challenges requires coordinated efforts to improve digital literacy, establish disclosure practices for AI-generated content, and develop regulatory frameworks that promote transparency and safeguard public trust in health communication.

This study presents several limitations, and its findings should be interpreted with caution. As with any cross-sectional design, causality cannot be established. Cultural factors, such as Brazilian beauty standards and participants’ familiarity with artificial intelligence, may have influenced responses and should be considered when generalizing the results. Moreover, the AI-generated images were created using a basic prompt (“Make a realistic perfect smile”), without optimization or advanced prompt engineering. This approach aimed to reflect typical real-world usage by non-expert users. Even with minimal input, the generated images reached a high level of realism, making it difficult for both professionals and laypeople to distinguish them from real clinical outcomes. Additionally, although the stimuli were carefully standardized, the number of images was intentionally restricted to nine to minimize participant fatigue and reduce cognitive overload, a strategy commonly adopted in visual perception research to enhance attentional engagement and response reliability^34^. Nevertheless, the use of a finite stimulus set may limit generalizability to other clinical presentations. Furthermore, familiarity with artificial intelligence technologies was not directly assessed among lay participants. Individuals with prior exposure to AI-generated imagery may demonstrate different detection abilities, representing a potential source of bias; however, such exposure may also reflect real-world conditions, where interaction with synthetic media is increasingly common. The study followed established standards in perceptual validation, including anonymized data collection, voluntary participation, and randomized image presentation^7,15,35^. The inclusion of laypeople, dental students, and dentists allowed for comparisons across different expertise levels, improving generalizability. A standardized photographic protocol also ensured consistency and visual accuracy^3,5^.

Future research should investigate the use of clinically detailed prompts and iterative generation to enhance anatomical realism. These findings highlight the need to examine how AI-generated visuals influence patient expectations and the potential spread of misinformation. Efforts should focus on improving prompt design, refining AI image control, and establishing ethical guidelines for transparent use of AI-generated content in clinical and public health communication.

## Conclusion

AI-generated smile images were frequently misclassified as real, while actual post-treatment outcomes were more accurately identified, regardless of treatment type among dentists, dental students and laypeople. Participants rated AI-generated smile images as more attractive than real orthodontic results.

## Supplementary Information

Below is the link to the electronic supplementary material.

Supplementary Material 1

## Data Availability

Data is provided within the manuscript or supplementary information file.
