# Supplementary material for: Perception of AI-generated smile versus real orthodontic treatment outcomes among dentists, students, and laypeople
Source: Sci Rep. 2026 Mar 21;16:14377. doi: 10.1038/s41598-026-41744-4 (PMC13144406; doi:10.1038/s41598-026-41744-4)
Supplement: Supplementary file 1 — Supplementary Material 1 [file 41598_2026_41744_MOESM1_ESM.docx]

*Supplementary 1. Images evaluated*


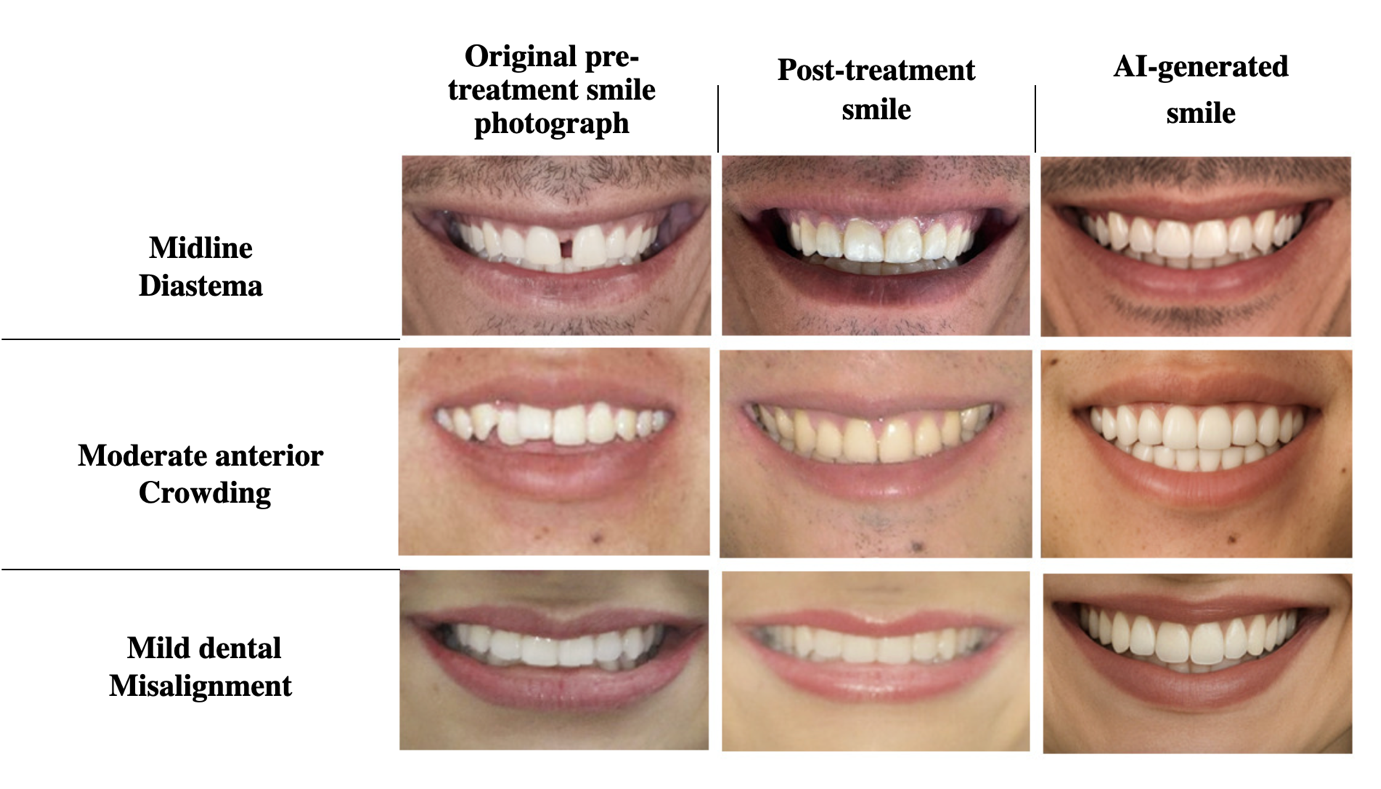

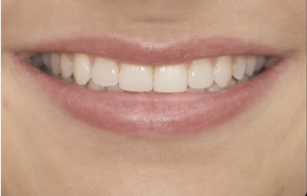

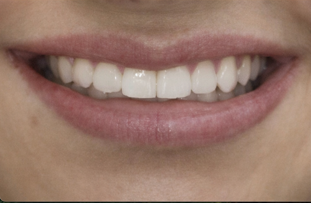

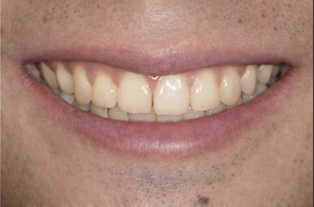


*Supplementary 2. Metrics of distinguishing real and AI-created images stratified by clinical experience*

|  | **ACCURACY (95% CI)** | **Sensitivity** | **Specificity** | **PPV** | **NPV** |
| --- | --- | --- | --- | --- | --- |
| **Dentists** | 0.626 (0.578 – 0.672) | 0.391 | 0.878 | 0.775 | 0.573 |
| **Dental Students** | 0.727 (0.678 – 0.773) | 0.489 | 0.982 | 0.967 | 0.642 |
| **Others** | 0.663 (0.630-0.695) | 0.412 | 0.930 | 0.861 | 0.598 |

*Table 3. Metrics of distinguishing real and AI-created images stratified by treatment type*

|  | **ACCURACY (95% CI)** | **Sensitivity** | **Specificity** | **PPV** | **NPV** |
| --- | --- | --- | --- | --- | --- |
| **Mild Dental Misalignment** | 0.686 (0.645 - 0.725) | 0.458 | 0.935 | 0.884 | 0.612 |
| **Midline Diastema** | 0.659 (0.617 - 0.699) | 0.407 | 0.921 | 0.843 | 0.599 |
| **Anterior Crowding** | 0.657 (0.616 - 0.698) | 0.404 | 0.927 | 0.855 | 0.594 |
